# Supplementary figures and images for: pMGF505-7R determines pathogenicity of African swine fever virus infection by inhibiting IL-1β and type I IFN production
Source: PLoS Pathog. 2021 Jul 26;17(7):e1009733. doi: 10.1371/journal.ppat.1009733 (PMC8341718; doi:10.1371/journal.ppat.1009733)

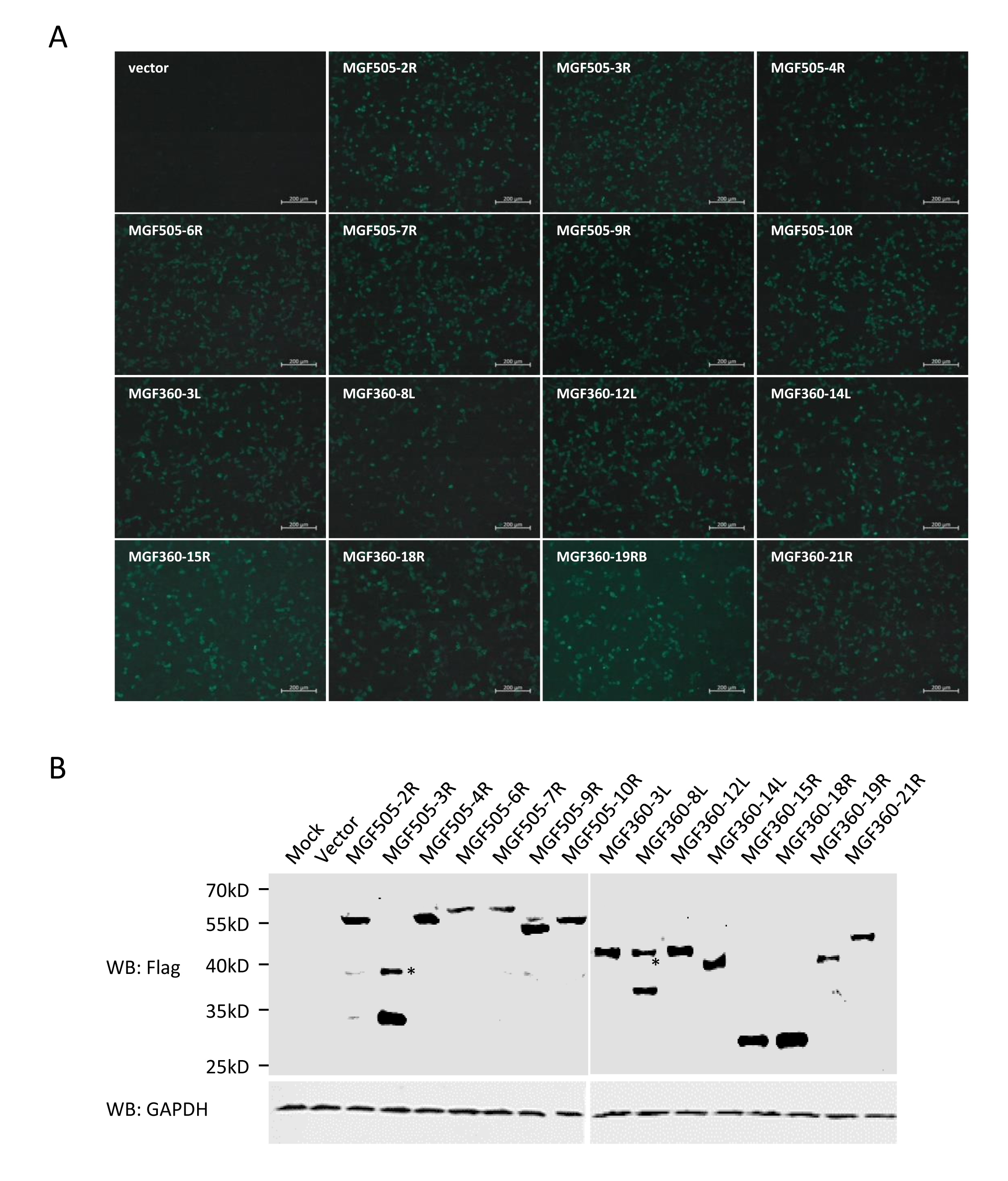

Supplement: S1 Fig — (A-B) HEK293T cells were transfected with plasmids expressing ASFV-encoded pMGFs in the presence of the iGLuc-based NLRP3 inflammasome system or IFN-β promoter reporter. The cells were probed with mouse anti-Flag mAb, and then observed by microscopy (A). The cells were detected by Western blot using anti-Flag mAb (B). * means a nonspecific band. (TIF) [file ppat.1009733.s001.tif]

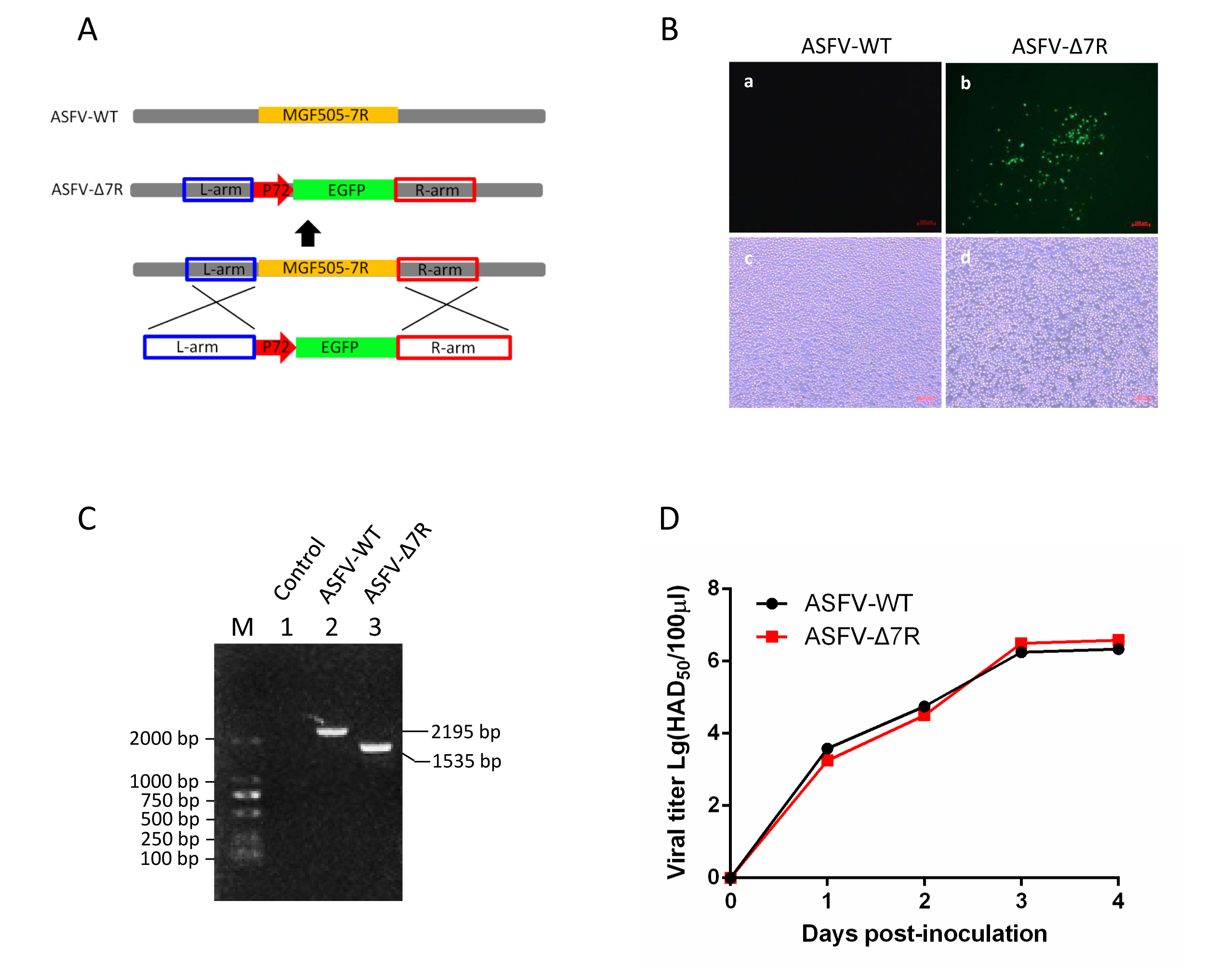

Supplement: S2 Fig — (A) Schematic representation of generation of ASFV-Δ7R. The MGF505-7R gene segment was replaced with the p72-EGFP reporter gene cassette. (B) PAMs were infected with ASFV (ASFV-WT) or ASFV-Δ7R. At 24 hpi, the cells were observed by microscope. (C) Agarose gel (1%) showing the result of the conventional PCR to amplify of the genomic segment containing the targeted gene. (D) Growth kinetics in PAMs for ASFV-Δ7R and ASFV-WT. PAMs were infected with ASFV-Δ7R or ASFV-WT (MOI = 0.01), and samples were taken from three independent experiments at the indicated time points and titrated. (TIF) [file ppat.1009733.s002.tif]

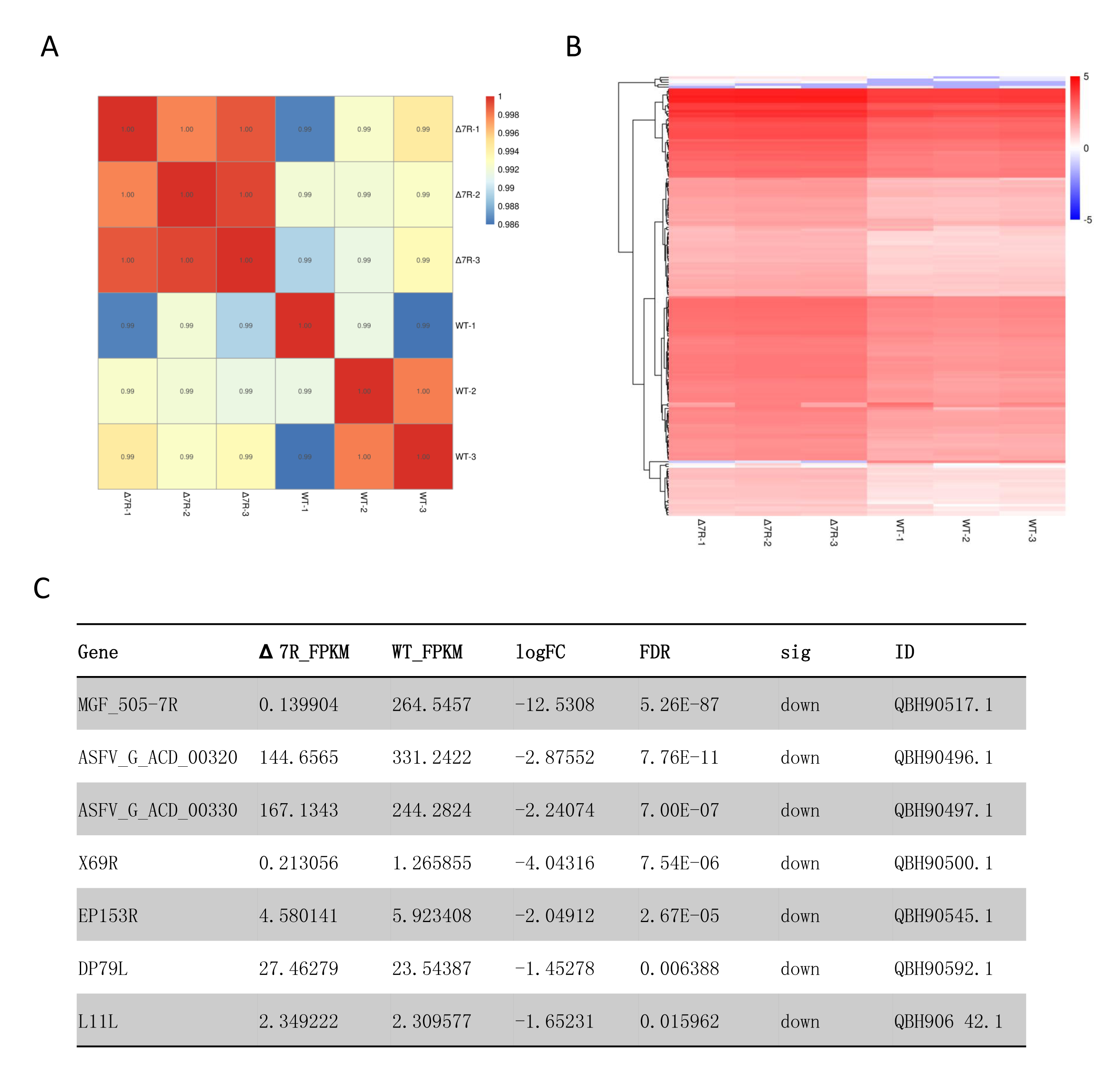

Supplement: S3 Fig — (A) Detection of RNA SEQ correlation among samples. (B) The heatmap of different categories in diverse pathological classes based on TCM Diagnostic System. Each row refers to a sample. The color variety showed the frequency of the targets in each category. (C) Gene expression levels were normalized using the FPKM method. Differentially expressed genes (DEGs) were analyzed by the edgR using fold change≥2 and FDR<0.05 thresholds. (TIF) [file ppat.1009733.s003.tif]

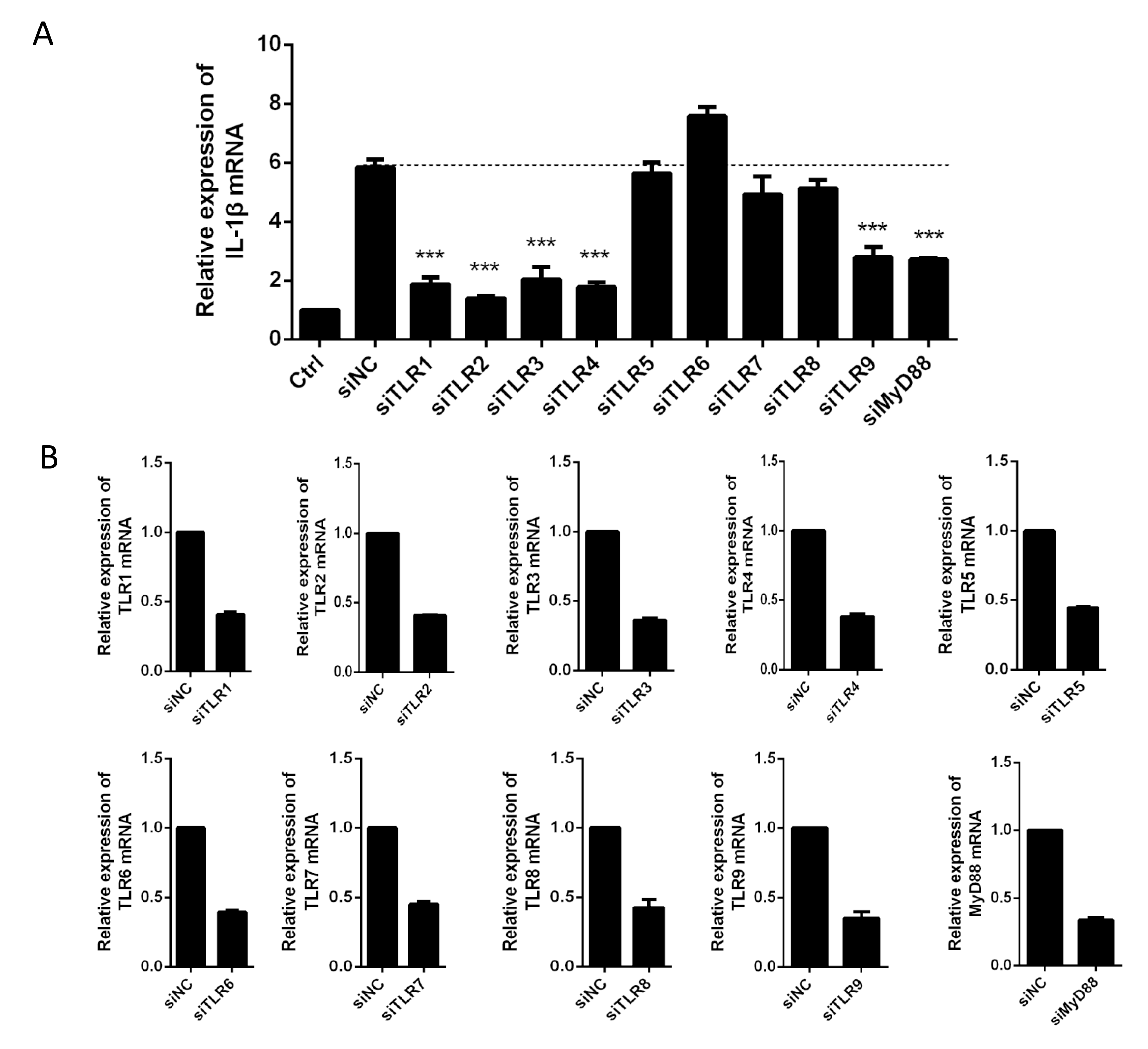

Supplement: S4 Fig — (A-B) PAMs were transfected with control siRNA (siNC) or siRNAs targeting each of the TLRs or MyD88. At 24 hpt, the cells were infected with ASFV-Δ7R at an MOI of 1 for another 24 h, then the mRNA levels of IL-1β were detected by qPCR (A). The mRNA levels of TLRs and MyD88 were detected to confirm the knockdown efficiencies mediated by the siRNAs (B). A p value of less than 0.05 was considered statistically significant. *p<0.05, **p<0.01, ***p<0.001. (TIF) [file ppat.1009733.s004.tif]

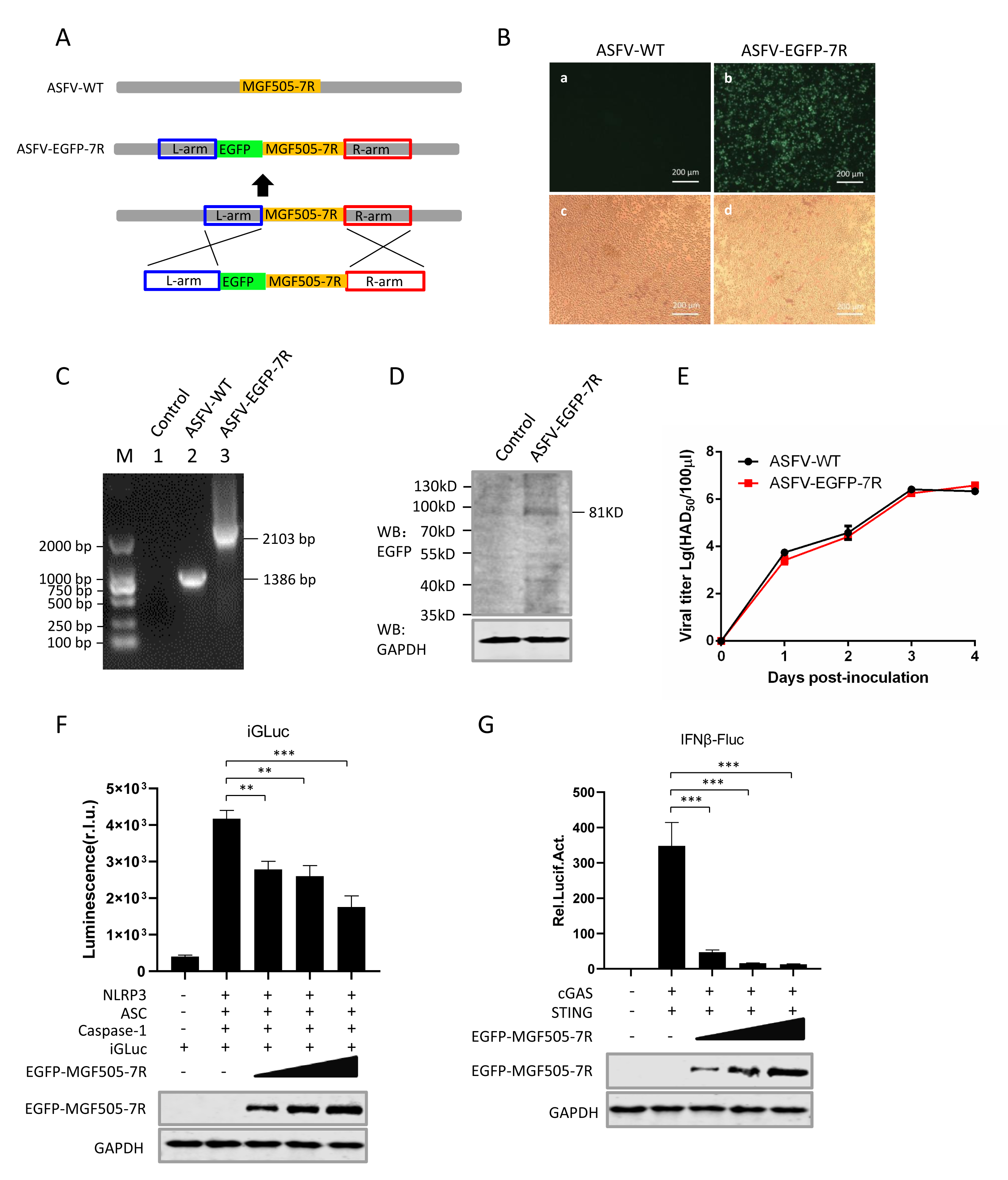

Supplement: S5 Fig — (A) Schematic representation of generation of ASFV with EGFP-tagged pMGF505-7R. The EGFP was inserted N-terminal of MGF505-7R. (B) PAMs were infected with ASFV (ASFV-WT) or ASFV-EGFP-7R. At 24 hpi, the cells were observed by microscope. (C) Agarose gel (1%) showing the result of the conventional PCR to amplify of the genomic segment containing the targeted gene. (D) PAMs were mock-infected or infected with ASFV-EGFP-7R (MOI = 1) for 36 h, and the expressing levels of EGFP-7R were detected by Western blotting. (E) Growth kinetics in PAMs for ASFV-EGFP-7R and ASFV-WT. PAMs were infected with ASFV-Δ7R or ASFV-WT (MOI = 0.01), and samples were taken from three independent experiments at the indicated time points and titrated. (F) HEK293T cells were transfected with increasing doses of a plasmid expressing pEGFP-MGF505-7R in the presence of the iGLuc-based NLRP3 inflammasome system, and the supernatants were assessed 24 hpt for luciferase activity. (G) HEK293T cells were co-transfected with increasing doses of a plasmid expressing EGFP-MGF505-7R and IFN-β promoter reporter for 24 h and then the cells were stimulated with poly(I:C) (1 μg) for another 12 h, the cells were then collected, and the luciferase activities were measured. A p value of less than 0.05 was considered statistically significant. *p<0.05, **p<0.01, ***p<0.001. (TIF) [file ppat.1009733.s005.tif]

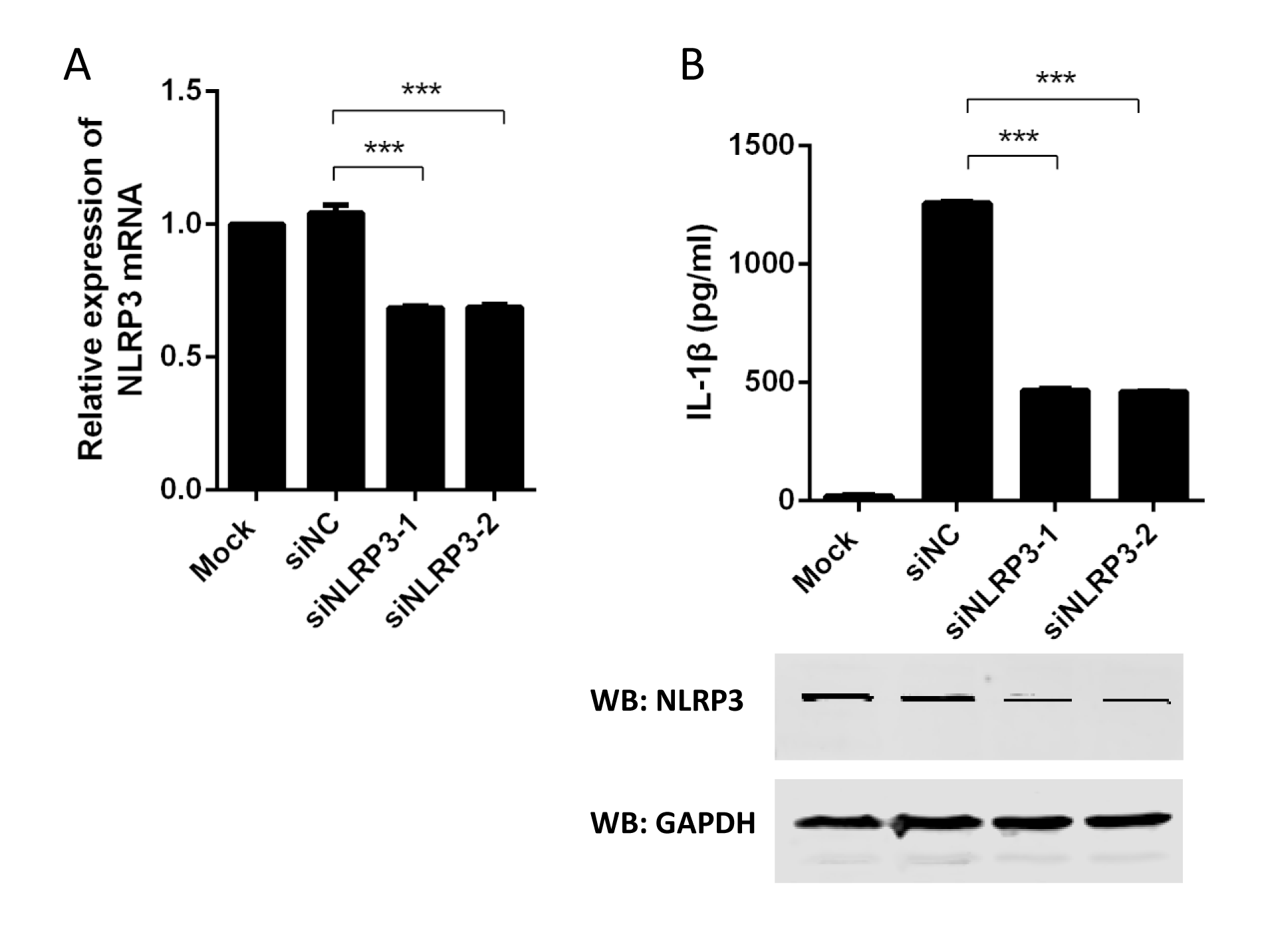

Supplement: S6 Fig — (A-B) PAMs were transfected with siRNAs targeting NLRP3. At 24 hpt, the cells were infected with ASFV-Δ7R at an MOI of 1 for another 24 h, then the mRNA levels of NLRP3 were detected by qPCR (A), the protein levels of NLRP3 were detected by Western blot and the secretion of IL-1β were detected by ELISA (B). A p value of less than 0.05 was considered statistically significant. *p<0.05, **p<0.01, ***p<0.001. (TIF) [file ppat.1009733.s006.tif]

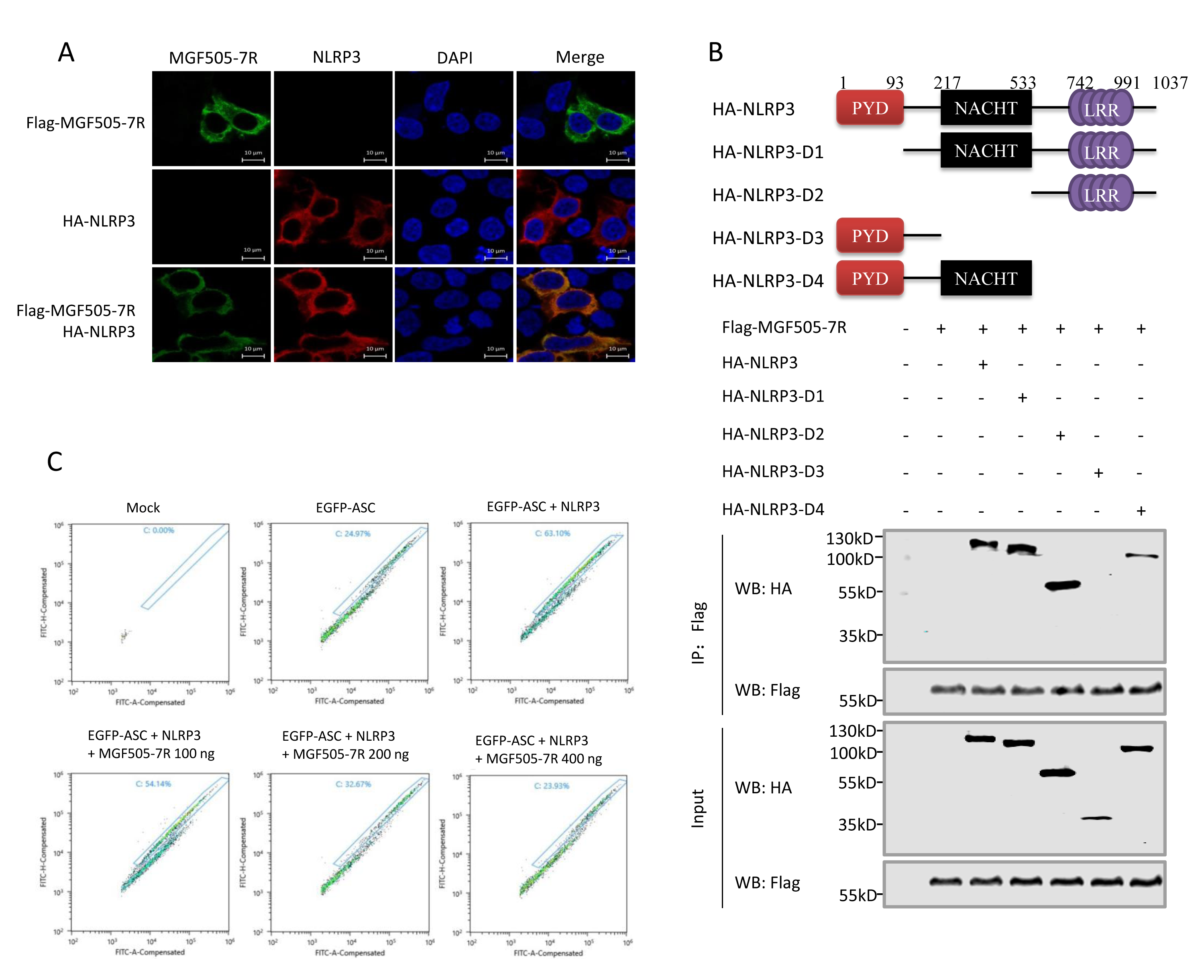

Supplement: S7 Fig — (A) Flag-pMGF505-7R and HA-NLRP3 were expressed individually or together in HEK293T cells. The cells were probed with rabbit anti-HA mAb and mouse anti-Flag mAb, stained with nucleus marker DAPI and then observed by confocal microscopy. (B) HEK293T cells were transfected with a plasmid encoding Flag-MGF505-7R alone or together with a plasmid encoding HA-NLRP3 or its truncated mutants. 48 hpt, cell lysates were collected and immunoprecipitated with anti-Flag antibody, followed by immunoblotting for HA-tagged NLRP3 and its truncated mutants. (C) HEK293T cells were transfected with plasmids expressing GFP-ASC alone, or GFP-ASC and HA-NLRP3, or GFP-ASC and HA-NLRP3 together with increasing amounts of a plasmid expressing Flag-pMGF505-7R. 24 hours post transfection, cells were harvested, fixed, and the fraction of cells containing ASC specks was quantified by flow cytometry. (TIF) [file ppat.1009733.s007.tif]
