# Supplementary material for: pMGF505-7R determines pathogenicity of African swine fever virus infection by inhibiting IL-1β and type I IFN production
Source: PLoS Pathog. 2021 Jul 26;17(7):e1009733. doi: 10.1371/journal.ppat.1009733 (PMC8341718; doi:10.1371/journal.ppat.1009733)
Supplement: S1 Table — (DOCX) [file ppat.1009733.s008.docx]

| S1 Table. Primers, siRNA and sgRNA used in this study. | | | |
| --- | --- | --- | --- |
| Resource | | **Sequence(5’-3’)** | **Reference** |
| Primer and sgRNA for ASFV-Δ7R | Upstream homologous arm of MGF505-7R | F: ggtaccgggccccccctcgagCTGGTAGAGCATGTAGTAAATGAGAAATC | this paper |
|  |  | R: atcgataccgtcgacctcgagTTTCCCTACCCAAATTAAAAAAAAA |  |
|  | Downstream homologous arm of MGF505-7R | F: aattcttttttttttggatccATTTCTGAATCAGTAAGCAATAGATAGATTT | this paper |
|  |  | R: cgctctagaactagtggatccTGGATCTTGAATCAGTGGTAGAATCT |  |
|  | sgRNA targeting MGF505-7R | GGAAGCTTGAGATTCTTACG | this paper |
|  | Identification of ASFV-Δ7R | F: AAGTTACTCACTAAAACACGA | this paper |
|  |  | R: ATACGGGGACCTATCTAAAAT |  |
| Primer for ASFV-Δ7R | Primer for EGFP amplification | F: tttttttaatttgggtagggaaaATGGTGAGCAAGGGCGAGG | this paper |
|  |  | R: ggtcctgaagggagaacatCTTGTACAGCTCGTCCATGCC |  |
|  | Primer for MGF505-7R amplification | F: GGCATGGACGAGCTGTACAAGatgttctcccttcaggacc | this paper |
|  |  | R: atttctgaatcagtaagcaatagTTAATACATGGCATACTCCAAAGCA |  |
|  | Primer for EGFP-MGF505-7R amplification | F: tttttttaatttgggtagggaaaATGGTGAGCAAGGGCGAGG | this paper |
|  |  | R: atttctgaatcagtaagcaatagTTAATACATGGCATACTCCAAAGCA |  |
|  | Identification of ASFV-EGFP-7R | F: GATATTCTTAAACTGGCAAC  R: AAGAGTAGAGTTGTATTTCAC | this paper |
| Primer for qPCR | Swine HPRT | F: GCCGAGGATTTGGAAAAGG | [1] |
|  |  | R: GCACACAGAGGGCTACGATG |  |
|  | Swine IL-1β | F: CCCAAAAGTTACCCGAAGAGG | [1] |
|  |  | R: TCTGCTTGAGAGGTGCTGATG |  |
|  | Swine TNF-α | F: ACCACGCTCTTCTGCCTACTGC | [1] |
|  |  | R: TCCCTCGGCTTTGACATTGGCTAC |  |
|  | Swine IL-6 | F: CTGCTTCTGGTGATGGCTACTG | [1] |
|  |  | R: GGCATCACCTTTGGCATCTT |  |
|  | Swine IFN-α | F: CTGCTGCCTGGAATGAGAGCC | [2] |
|  |  | R: TGACACAGGCTTCCAGGTCCC |  |
|  | Swine IFN-β | F: AGCACTGGCTGGAATGAAACCG | [2] |
|  |  | R: CTCCAGGTCATCCATCTGCCCA |  |
|  | ASFV genomic copies | F: CTGCTCATGGTATCAATCTTATCGA | [3] |
|  |  | R: GATACCACAAGATCAGCCGT |  |
|  |  | Probe: FAM-CCACGGGAGGAATACCAACCCAGTG-TAMRA |  |
|  | Swine TLR1 | F: TGGAAGAGGTCAGGATCACC | [4] |
|  |  | R: GATGGCAAAATGGAAGATGC |  |
|  | Swine TLR2 | F: ACGGACTGTGGTGCATGAAG | [4] |
|  |  | R: GGACACGAAAGCGTCATAGC |  |
|  | Swine TLR3 | F: GACCTCCCGGCAAATATAAC | [4] |
|  |  | R: GGGAGACTTTGGCACAATTC |  |
|  | Swine TLR4 | F: TGTGCGTGTGAACACCAGAC | [4] |
|  |  | R: AGGTGGCGTTCCTGAAACTC |  |
|  | Swine TLR5 | F: GGACTTGACAACCTCCAGATTCT | [4] |
|  |  | R: AGGAACCTGAATGTTTGGTCCT |  |
|  | Swine TLR6 | F: GGGGAACCCTAATCCAGTTC | [4] |
|  |  | R: AGCTGCGAGAGAAAGCTGAT |  |
|  | Swine TLR7 | F: CGGTGTTTGTGATGACAGAC | [4] |
|  |  | R: AACTCCCACAGAGCCTCTTC |  |
|  | Swine TLR8 | F: CACATTTGCCCGGTATCAAG | [4] |
|  |  | R: TGTGTCACTCCTGCTATTCG |  |
|  | Swine TLR9 | F: GGCCTTCAGCTTCACCTTGG | [4] |
|  |  | R: GGTCAGCGGCACAAACTGAG |  |
|  | Swine MyD88 | F: GGCAGCTGGAACAGACCAA | [5] |
|  |  | R: GGTGCCAGGCAGGACATC |  |
| siRNA | Targeting swine TLR1 | cccacaaagttacatctat | [5] |
|  | Targeting swine TLR2 | gcccttcctacacacttta | [5] |
|  | Targeting swine TLR3 | gcttaagtgtgattggtaa |  |
|  | Targeting swine TLR4 | gagcttaatgtggctcaca | [6] |
|  | Targeting swine TLR5 | gccttcaacaagataaaca | [5] |
|  | Targeting swine TLR6 | gcccaaacctgtagaatat | [5] |
|  | Targeting swine TLR7 | ccagaaggcagcagattaa | [7] |
|  | Targeting swine TLR8 | gctggaagacaaccagtta | [7] |
|  | Targeting swine TLR9 | gcctctccttactctccaa | [8] |
|  | Targeting swine MyD88 | atgcctgagcattttgatg | [9] |
|  | Targeting swine NLRP3-1 | GCATCTATTCTGCAAGCTA | this paper |
|  | Targeting swine NLRP3-2 | GCACCCGAACTGCAAGCTT | this paper |

1. Li J, Hu L, Liu Y, Huang L, Mu Y, Cai X, et al. DDX19A Senses Viral RNA and Mediates NLRP3-Dependent Inflammasome Activation. J Immunol. 2015;195(12):5732-49. Epub 2015/11/06. doi: 10.4049/jimmunol.1501606. PubMed PMID: 26538395.

2. Huang L, Liu Q, Zhang L, Zhang Q, Hu L, Li C, et al. Encephalomyocarditis Virus 3C Protease Relieves TRAF Family Member-associated NF-kappaB Activator (TANK) Inhibitory Effect on TRAF6-mediated NF-kappaB Signaling through Cleavage of TANK. J Biol Chem. 2015;290(46):27618-32. Epub 2015/09/13. doi: 10.1074/jbc.M115.660761. PubMed PMID: 26363073; PubMed Central PMCID: PMCPMC4646013.

3. King DP, Reid SM, Hutchings GH, Grierson SS, Wilkinson PJ, Dixon LK, et al. Development of a TaqMan PCR assay with internal amplification control for the detection of African swine fever virus. J Virol Methods. 2003;107(1):53-61. Epub 2002/11/26. doi: 10.1016/s0166-0934(02)00189-1. PubMed PMID: 12445938.

4. Hamonic G, Pasternak JA, Forsberg NM, Kaser T, Wilson HL. Expression of pattern recognition receptors in porcine uterine epithelial cells in vivo and in culture. Vet Immunol Immunopathol. 2018;202:1-10. Epub 2018/08/07. doi: 10.1016/j.vetimm.2018.06.006. PubMed PMID: 30078581.

5. Chen Y, Liu T, Langford P, Hua K, Zhou S, Zhai Y, et al. Haemophilus parasuis induces activation of NF-kappaB and MAP kinase signaling pathways mediated by toll-like receptors. Mol Immunol. 2015;65(2):360-6. Epub 2015/03/04. doi: 10.1016/j.molimm.2015.02.016. PubMed PMID: 25733389.

6. Wachi S, Kanmani P, Tomosada Y, Kobayashi H, Yuri T, Egusa S, et al. Lactobacillus delbrueckii TUA4408L and its extracellular polysaccharides attenuate enterotoxigenic Escherichia coli-induced inflammatory response in porcine intestinal epitheliocytes via Toll-like receptor-2 and 4. Molecular nutrition & food research. 2014;58(10):2080-93. Epub 2014/07/06. doi: 10.1002/mnfr.201400218. PubMed PMID: 24995380.

7. Bi J, Song S, Fang L, Wang D, Jing H, Gao L, et al. Porcine reproductive and respiratory syndrome virus induces IL-1beta production depending on TLR4/MyD88 pathway and NLRP3 inflammasome in primary porcine alveolar macrophages. Mediators Inflamm. 2014;2014:403515. Epub 2014/06/27. doi: 10.1155/2014/403515. PubMed PMID: 24966466; PubMed Central PMCID: PMCPMC4055429.

8. Cao L, Ge X, Gao Y, Ren Y, Ren X, Li G. Porcine epidemic diarrhea virus infection induces NF-kappaB activation through the TLR2, TLR3 and TLR9 pathways in porcine intestinal epithelial cells. J Gen Virol. 2015;96(Pt 7):1757-67. Epub 2015/03/31. doi: 10.1099/vir.0.000133. PubMed PMID: 25814121.

9. Qin Y, Li H, Qiao J. TLR2/MyD88/NF-kappaB signalling pathway regulates IL-8 production in porcine alveolar macrophages infected with porcine circovirus 2. J Gen Virol. 2016;97(2):445-52. Epub 2015/11/20. doi: 10.1099/jgv.0.000345. PubMed PMID: 26581603.

## Reference
