# Supplementary material for: pMGF505-7R determines pathogenicity of African swine fever virus infection by inhibiting IL-1β and type I IFN production
Source: PLoS Pathog. 2021 Jul 26;17(7):e1009733. doi: 10.1371/journal.ppat.1009733 (PMC8341718; doi:10.1371/journal.ppat.1009733)
Supplement: S2 Table — (DOCX) [file ppat.1009733.s009.docx]

S2 Table. The DNA sequence covering the modified region of ASFV

| Recombinant ASFV | Sequence(5’-3’) |
| --- | --- |
| ASFV-Δ7R | actgtttttagagtgtttagaaattgctgatgagaaggagtttcctgatattaaaagtattgtgagtgaatatattaactacttgtttactgcaggagctattaccaaggaagaaatcatgcaagcctatgatgctttagagtagccatgtattaacattctgaaagtagaataaaatatactatatactaaaaaccaaattagccatttttaactatcttcttcttaaaaactctggataaaaatttatttttttttaatttgggtagggaaa**ATAACTTCGTATAATGTATGCTATACGAAGTTATGGGTCGCCGGAGGAAAAGTCAAAAGGGGCAGGTAGTTCATACACCAAAAAGTTTTTTTTTTCTGCCAGCAAGAGCGTGTCAATAATTTTAAGCTGATCGTTAATTAATTTTTGGTTTAACTCTTTGTTATTATCAAGATCCTTCGCATAAACCGCCATATTTAATAAAAACAATAAATTATTTTTATAACATTATATATGGTGAGCAAGGGCGAGGAGCTGTTCACCGGGGTGGTGCCCATCCTGGTCGAGCTGGACGGCGACGTAAACGGCCACAAGTTCAGCGTGTCCGGCGAGGGCGAGGGCGATGCCACCTACGGCAAGCTGACCCTGAAGCTGATCTGCACCACCGGCAAGCTGCCCGTGCCCTGGCCCACCCTCGTGACCACCCTGGGCTACGGCCTGCAGTGCTTCGCCCGCTACCCCGACCACATGAAGCAGCACGACTTCTTCAAGTCCGCCATGCCCGAAGGCTACGTCCAGGAGCGCACCATCTTCTTCAAGGACGACGGCAACTACAAGACCCGCGCCGAGGTGAAGTTCGAGGGCGACACCCTGGTGAACCGCATCGAGCTGAAGGGCATCGACTTCAAGGAGGACGGCAACATCCTGGGGCACAAGCTGGAGTACAACTACAACAGCCACAACGTCTATATCACCGCCGACAAGCAGAAGAACGGCATCAAGGCCAACTTCAAGATCCGCCACAACATCGAGGACGGCGGGGTGCAGCTCGCCGACCACTACCAGCAGAACACCCCCATCGGCGACGGCCCCGTGCTGCTGCCCGACAACCACTACCTGAGCTACCAGTCCGCCCTGAGCAAAGACCCCAACGAGAAGCGCGATCACATGGTCCTGCTGGAGTTCGTGACCGCCGCCGGGATCACTCTCGGCATGGACGAGCTGTACAAG**TGATAAATAACTTCGTATAATGTATGCTATACGAAGTTATatttctgaatcagtaagcaatagatagattttagaatatgctgtattaagttagtttctgaataagtaattaatagatagattttagtttatgtaaaaatgttaacatttgttcataagttttagataccattttagagttactttttt  (RED: p72 promoter; **Green: EGFP)** |
| ASFV-GFP-7R | gctttagagtagccatgtattaacattctgaaagtagaataaaatatactatatactaaaaaccaaattagccatttttaactatcttcttcttaaaaactctggataaaaatttatttttttttaatttgggtagggaaa**ATGGTGAGCAAGGGCGAGGAGCTGTTCACCGGGGTGGTGCCCATCCTGGTCGAGCTGGACGGCGACGTAAACGGCCACAAGTTCAGCGTGTCCGGCGAGGGCGAGGGCGATGCCACCTACGGCAAGCTGACCCTGAAGCTGATCTGCACCACCGGCAAGCTGCCCGTGCCCTGGCCCACCCTCGTGACCACCCTGGGCTACGGCCTGCAGTGCTTCGCCCGCTACCCCGACCACATGAAGCAGCACGACTTCTTCAAGTCCGCCATGCCCGAAGGCTACGTCCAGGAGCGCACCATCTTCTTCAAGGACGACGGCAACTACAAGACCCGCGCCGAGGTGAAGTTCGAGGGCGACACCCTGGTGAACCGCATCGAGCTGAAGGGCATCGACTTCAAGGAGGACGGCAACATCCTGGGGCACAAGCTGGAGTACAACTACAACAGCCACAACGTCTATATCACCGCCGACAAGCAGAAGAACGGCATCAAGGCCAACTTCAAGATCCGCCACAACATCGAGGACGGCGGGGTGCAGCTCGCCGACCACTACCAGCAGAACACCCCCATCGGCGACGGCCCCGTGCTGCTGCCCGACAACCACTACCTGAGCTACCAGTCCGCCCTGAGCAAAGACCCCAACGAGAAGCGCGATCACATGGTCCTGCTGGAGTTCGTGACCGCCGCCGGGATCACTCTCGGCATGGACGAGCTGTACAAGatgttctcccttcaggacctctgtcggaagaacaccttcttccttccaagtgattttagcaagcataccctgcatttgctggggttatactggaaggggcatggatctatccaaaggataaagaatgatggtgtgcttatagagcatgatcttactctttccatcaatgaagccttaattcttgcaggagaagagggaaacaatgaagtagtaaagctcttgttactatgggaaggaaatcttcattatgccatcataggagctttgaggactgagaactataacctagtatgtgagtaccatagtcaaattcaggactggcatgttctcctccctttgattcaagatccagaaacattcgaaaaatgtcatgatttaagccttgaatgtgatctttcatgccttctccaacatgctgtaaaatataacatgctttcgat**  (**Green: EGFP; Black bold: MGF505-7R**) |
