# Supplementary material for: pMGF505-7R determines pathogenicity of African swine fever virus infection by inhibiting IL-1β and type I IFN production
Source: PLoS Pathog. 2021 Jul 26;17(7):e1009733. doi: 10.1371/journal.ppat.1009733 (PMC8341718; doi:10.1371/journal.ppat.1009733)
Supplement: Attachment — Submitted filename: (Version 15) Response to reviewer 20210530.docx [file ppat.1009733.s010.docx]

**Response letter**

**Responses to Reviewer #1:**

**Summary and Major Issues:**

The manuscript by Li et al., contributes interesting data about the determination on how ASFV infection impaired the immune response by inducing low levels of IL-1b and type I IFNs. It also identifies some of the ASFV genes that are responsible for the modulation of the immune response during the ASFV infection. Authors demonstrate this modulation by doing different experiments in vitro, by using PAMs, and in vivo on pigs. This contribution on the field of ASFV research is important not only to better understand the pathogenesis of ASFV but also in the study of potential vaccine candidates.
Overall, the paper has good quality for publication.
The hypothesis in the paper have been mostly answered by experimental procedures which confirm the hypothesis. The methodology and results are well described. The results are clear and answered the topic studied. The paper is well written and the bibliography is up to date.

As a suggestion, it could have been interesting to study the consequences of the IKK alpha interaction downstream of the interaction. It could be interesting to analyze p65 translocation or the phosphorylation of IKB alpha.

The research topic is well described and the methodology is well planned from the beginning (cell culture) to the end of the study (animal models). Finally, all results are accompanied by statistical data.
**Question 1:** As a suggestion, it could have been interesting to study the consequences of the IKK alpha interaction downstream of the interaction. It could be interesting to analyze p65 translocation or the phosphorylation of IKB alpha.

Authors’ response:

Thank you for your suggestion. According to your suggestion, we tested the phosphorylation of IκBα and p65 translocation in the condition of ASFV infection. We confirmed that pMGF505-7R interacted with IKKα under the condition of overexpression and ASFV infection. In addition, we demonstrated that pMGF505-7R inhibited IκBα phosphorylation and p65 translocation to nucleus. Please see the Fig. 6 and the text (lines 264-272) in the revised manuscript.

**Question 2:** Luciferase assays should clarify if authors include empty vector with just the tag (MGF505-7R) as a control. If not, authors should include it necessarily.

Authors’ response:

For the luciferase assays, the total amount of DNA (500 ng) in each sample was kept constant by supplementation with the empty vector. The empty vector is the vector used to construct the expression plasmid (MGF505-7R), as the control of the experiment. Please see the changes (lines 523-524) in Materials and Methods in the revised manuscript.

**Question 3:** Immunoprecipitation assays should include the empty vector with the tag as a control.

Authors’ response:

For immunoprecipitation assays, the total amount of DNA in each sample was kept constant by supplementation with the empty vector. According to your suggestion, we provided more detailed information (lines 554-557) in Materials and Methods. And we repeated the experiment and obtained the same results. Please see the changes (Fig 6D, Fig 7B and Fig S7B) in the revised manuscript.

**Question 4:** In the screening to select optimal MGF some controls are missing. This reviewer find that the Supplementary IFI are not the optimal approach to test if expression was similar with all the constructions. It is necessary to include a robust analysis of the expression levels between plasmids given the very close levels of luminescence inhibition found.
Authors’ response:

Thank you for your suggestion. We repeated the experiment and found that the trend of the results is consistent with the previous. And we added the WB result for the expression levels of pMGFs. Please see the results in the revised manuscript (supporting information, Fig. S1B).

**Question 5:** More level of detail would be desirable for Materials and Methods section. Concentrations of plasmids or stimulators are not easy to find. We suggest to include a Table in M&M with all plasmids and concentrations used.

Authors’ response:

Thank you for your suggestion. In different experiments, the plasmids or stimulators were used largely in different concentrations, therefore, it is difficult to use a table to describe them all. So, we provided more detailed information concerning the concentrations of plasmids or stimulators used in the research in Materials and Methods section and Figure Legends. For examples, LPS (100 ng/ml), Nigericin (5 µM), and the iGLuc-based NLRP3 inflammasome system (100 ng iGLuc, 10 ng pCAGGS-caspase-1, 10 ng pCAGGS-ASC, and 12.5 ng pCAGGS-NLRP3) were used in the study.

Please see the changes (For example, Lines 866-870) in the revised manuscript.

**Minor Issues:**

**Question 1:** As minor revisions, please check figure 5 A and B where I suggest include the name of each condition in the experiment under each column (similar to Figure 5 C and D for clarification).

Authors’ response:

Thank you for your suggestion. We corrected it in the revised manuscript. Please see the Fig. 5 in the revised manuscript.

**Responses to Reviewer #2:**

**Summary**

The manuscript describes some new characteristics of ASFV MGF505 members in modulating IL-1β and type I IFN production in infected cells and pigs. Among the ASFV MGF505 members, the authors have found the strongest inhibitory effect of MGF505-7R gene indicating its potential in developing a new attenuated vaccine against ASF. Subsequently, the authors have studied signaling pathways and localization of the ASFV MGF505-7R. They have shown that MGF505-7R mode of action is primarily via by the interaction with IKKα complex which leads to NF-κB inhibition and bounding to NLRP3, leading to decreased IL-1β production.
It is worth noting that several studies have already described similar data regarding the role of ASFV MGF505 in IFN inhibition. In particular, Dan Li et al (2021) have presented identical results using recombinant ASFV-Δ7R (African Swine Fever Virus MGF-505-7R Negatively Regulates cGAS-STING-Mediated Signaling Pathway).
Remarkable results have been generated using recombinant ASFV lacking the MGF505-7R gene (ASFV-Δ7R) and protective potential of ASFV-Δ7R has been discovered (60% of animals survived the challenge).

**Question 1:** It is worth noting that several studies have already described similar data regarding the role of ASFV MGF505 in IFN inhibition. In particular, Dan Li *et al* (2021) have presented identical results using recombinant ASFV-Δ7R (African Swine Fever Virus MGF-505-7R Negatively Regulates cGAS–STING-Mediated Signaling Pathway).
Authors’ response:

During review of this manuscript, the paper (Li *et al.*, J Immunol, 2021) characterizing the role of ASFV MGF-505-7R in negatively regulating cGAS-STING pathway by promoting ULK1-mediated STING degradation was published. Both researches represent totally independent work. Although Li *et al.* also characterized ASFV MGF-505-7R as a key negative regulator of the host innate immune system and identified the underling mechanism, it does not compromise the novelty of the paper too much as we uncovered totally different mechanism by which ASFV antagonizes IL-1β and type I IFN production. Our data, beyond those previously published, still have obvious novelties, including the following aspects:

1. Inflammatory factors are key components of host innate immunity and can pose a strong anti-viral effect against viruses. IL-1β, a potent pleiotropic proinflammatory cytokine produced predominantly by monocytes, macrophages, and lymphocytes, plays pivotal roles in regulating innate immune responses and instructing adaptive immune responses. In the study, we found that ASFV infection induces low levels of IL-1β production through TLRs/MyD88 pathway and NLRP3 inflammasome in PAMs (Fig. 1, Fig. S4 and Fig. S6). Also, we found that ASFV antagonizes IL-1β production even in the presence of strong inducers such as LPS and Nigericin for IL-1β and type I IFN. To explore the underling mechanism, we screened members of pMGFs for regulation of IL-1β production and found that pMGF505-7R shows the strongest inhibitory effect (Fig 3A). Furthermore, we demonstrated that pMGF505-7R interacts with the IKKα complex to inhibit NF-κB activation, leading to reduced pro-IL-1β transcription and production (Fig1, 4 and 6); meanwhile, pMGF505-7R binds to NLRP3 to inhibit NLRP3 inflammasome assembly, leading to decreased IL-1β production (Fig 7). These results are reported for the first time so far.

2. Type I IFNs are critical host immune mediators for inhibiting viral infections. In this study, we found that ASFV infection induces low levels of type I IFNs in PAMs and pigs (Fig. 1 and 10). Mechanistically different from the one reported by Li et al. in which pMGF505-7R directly targets STING for degradation in the cGAS-STING signaling pathway by recruiting ULK1, in this research we showed that pMGF505-7R inhibits type I IFNs production by directly binding to IRF3 to block its nuclear translocation (Fig. 8). These data also represent novel discovery of the mechanism by which ASFV utilizes to block type I IFN signaling.

3. In animal experiment, Li *et al.* showed that the virulence of ASFV lacking MGF505-7R (ASFV-Δ7R) is dramatically reduced and it is safe to piglets. This conclusion may mislead the researchers who are designing attenuated live vaccines (ALVs) for ASFV as our data showed that ASFV-Δ7R still has moderate virulence. Therefore, it is more urgent to publish our results to provide more information about ASFV studies. In our study, pigs were challenged intramuscularly (i.m.) with ASFV-Δ7R or ASFV-WT at a dosage of 10^3^ or 10^5^ HAD_50_ (Fig 9). The body temperatures of all animals increased gradually, peaked at 41.7°C by day 9 (Fig 9A), and over 60% of pigs challenged with the attenuated ASFV-Δ7R survived (Fig 9B). It is noteworthy that the viral DNA copies in the ASFV-Δ7R-inoculated pigs decreased 2 to 3 orders of magnitude (Fig 9C). We also found that the attenuated ASFV-Δ7R induced higher levels of type I IFN and IL-1β production in pigs (Fig 10), which may help explain the decreased virulence of ASFV-Δ7R. These data provide more detailed information for attenuated ASFV-Δ7R, which may help researchers for designing effective vaccines against ASFV.

Taken together, we sincerely hope that our findings can provide more information for ASFV researchers.

**Major Issues**

**Question 1:** Several studies have shown that the ASFV has an arsenal of immunomodulatory proteins and one of the most sophisticated system of immune evasion among viruses. In order to decipher such complex interactions between cell and virus proteins, single virus protein analysis unable to reveal the whole specter of multimodal actions that likely to happen in infected cells or organism.

Authors’ response:

It is good question. During virus infection of the host cells or organism, there exists a series of complex virus-host interactions which involves many viral and host proteins. Therefore, experimentally deciphering all of these complex interactions simultaneously would be rather difficult and intimidating. During the past several years, our lab tried to use different screening systems to identify ASFV virulence gene involved in processes such as apoptosis, innate immunity, inflammatory responses and autophagy.

In this study, we screened the pMGFs members of ASFV for regulation of IL-1β and type I IFNs and found many members showed obvious inhibitory effects. In order to uncover the mechanism by which pMGF members inhibit IL-1β and type I IFNs production and thus explain how ASFV antagonizes host immunity, it is difficult and unrealistic for us to study all of these pMGF members simultaneously. Therefore, we selected one member of the strongest effect (MGF505-7R) for mechanistic study. We found that ASFV pMGF505-7R is a multifunctional protein, which inhibited IL-1β and type I IFN production in PAMs upon viral infection. In addition, we demonstrated that pMGF505-7R interacted with IKKα complex to inhibit NF-κB activation and also bound to NLRP3 to inhibit inflammasome formation, leading to decreased IL-1β production. Moreover, pMGF505-7R inhibited the nuclear translocation of IRF3 to block type I IFN production. Importantly, the virulence of ASFV-Δ7R is reduced in piglets compared with its parental ASFV due to induction of higher IL-1β and type I IFN *in vivo*. Our findings provide solid evidence to understand the multifunctional property of MGF505-7R and its involvement in viral pathogenesis, which serves as an example of viral virulence genes involved in modulating host immunity through virus-host protein interactions and might provide insights about the functional mechanisms for other pMGF members.

**Question 2:** All MGF505 members have shown an inhibitory effect on IL-1β production. However, the authors have chosen just one protein (MGF505-7R) with the strongest potential. The would be interesting to see whether the MGF505 proteins have a complementary effect on the IL-1β and type I IFN production.

Authors’ response:

It is a good question.

In the study, we believe that pMGF505-7R has the inhibitory effect on IL-1β and type I IFN production. On the one hand, ASFV-Δ7R infection induced higher type I IFN and IL-1β compared with ASFV-WT *in vitro* and *in vivo*. On the other hand, we analyzed the transcriptome of PAMs infected with ASFV-Δ7R and ASFV-WT and found that the expression levels of other genes from MGF360 and MGF505 families did not significantly changed, except MGF505-7R (Fig. S3).

According to your suggestion, we tested the complementary effect between other pMGF505 members and pMGF505-7R *in vitro* (HEK293T cells), and found that two MGF505 proteins don’t show stronger inhibitory effect than just one when the total amount of transfected plasmid was the same. But in the very limited time, we could not construct and test a new ASFV virus with at least two pMGF505 genes deleted. It will be interesting to test whether ASFV with two or more pMGF505 genes deleted shows more reduced virulence compared with ASFV-Δ7R; if so, then it may be a viable strategy to develop novel live attenuated vaccines against ASFV.

**Question 3:** The authors claim that 60% of pigs infected with the ASFV-Δ7R survived the challenge. I would recommend to add the comparative data between survived animals and those who succumbed the disease (40%) in the context of IL-1β and type I IFN level.

Authors’ response:

It’s a good question.

We tried to compare the difference in IL-1β and type I IFN level between survived and dead animals, but we didn't find significant difference due to the small sample size of data. In the current situation, we analyzed the original data of animal experiments, please see the following Figures.

**Minor Issues**

**Question 1:** Line 76 - ASF is arguably the most dangerous swine disease, but not the most serious viral diseases as stated in the manuscript. Please attenuate the statement.

Authors’ response:

We corrected it as “African swine fever (ASF) is one of the most lethal viral diseases affecting both domestic and wild swine.” (line 66) in the revised manuscript. Thank you!

**Question 2:** Line 86 - Please edit lower case letters "innate"

Authors’ response:

We corrected it in the revised manuscript. Thank you!

**Question 3:** Line 112- Please edit a capitalized word "Pathway"

Authors’ response:

We corrected it in the revised manuscript. Thank you!

**Question 4:** Line 313 - please rephrase the sentence.

Authors’ response:

Thank you for your suggestion. We reedited the sentence (line 345-346) as following:

Pigs inoculated with ASFV-WT began to die from day 8 after virus inoculation, and all died on day 10.

**Question 5:** Fig.1 - I assume that ASFV-WT has been used here. Please use the same virus title along the manuscript.

Authors’ response:

Thank you for your kindness and correction.

In this study, the ASFV HLJ/18 strain (strain) is referred to as ASFV-WT, and ASFV-Δ7R was generated from the ASFV HLJ/18 strain by deleting MGF505-7R gene using CRISPR/Cas9. We have revised the figure legends and provided more information to make the statement clearer in the revised manuscript.

**Question 6:** MOI 0.5 of ASFV has been used for animal trial, however MOI 0.01, 0.1 and 1 have been tested in vitro. Please provide some rationale for this.

Authors’ response:

We had previously established ASFV infection animal models using ASFV HLJ/18, the same strain we used in this study, to infect SPF piglets. According to the animal infection model established, we chose 10^3^ HAD_50_/piglet or 10^5^ HAD_50_/piglet virus to inoculate pigs. Please see the following reference.

Zhao D, Liu R, Zhang X, Li F, Wang J, Zhang J, et al. Replication and virulence in pigs of the first African swine fever virus isolated in China. Emerg Microbes Infect. 2019;8(1):438-47. Epub 2019/03/23. doi: 10.1080/22221751.2019.1590128

**Question 7:** Figure. 5C - No difference in genome copies between ASFV-WT and ASFV -d7R ha seen found in infected cells. However, decreased amount of ASFV0d7R genomes has been detected in infected animals. Please elaborate this founding in the discussion section.

Authors’ response:

Thank you for your suggestion. We discussed the difference about the genome copies of MGF505-7R and ASFV-WT *in vitro* and *in vivo* (line 468-473) in the discussion section in the revised manuscript.

**Question 8:** Fig.7. C - is hard to interpret and need more clarification.

Authors’ response:

According to the suggestions from you and the other reviewer, we reorganized the results in figure 7 in the revised manuscript. The Fig. 7C in the previous manuscript was moved to Fig. S7A in the revised version and replaced with the Co-IP result of endogenous NLRP3 and ASFV-EGFP-7R. We added more clarified figure legend for Fig. S7A, please see it in the revised Supporting Information document.

**Question 9:** Fig.11 describes the signaling pathway and a role of MGF-7R in it. The figure will benefit if other viral proteins involved in the same pathway will be noted.

Authors’ response:

According to your suggestion, we reviewed the references about the ASFV-encoded proteins involved in the inflammatory and interferon signaling pathways. We found that several ASFV-encoded proteins are reported to regulate type I IFN signaling pathway and NF-κB reporter activation. However, most studies are at the phenomenal stage, without mechanism or in-depth study. Therefore, we selected one protein (pMGF-505-7R) with mechanism and showed it functions in the Fig.11. Please see the changes in the revised manuscript.

| **Immune Evasion Strategies** | **Genes**  **(Encoding proteins)** | **Modes of functions** |
| --- | --- | --- |
| Inhibit apoptosis | A179L | A homolog of Bcl-2, interacting with BH3-only proteins |
|  | A224L | A homolog of IAP, inhibiti ng caspase-3 activation and activating NF-κB |
|  | EP153R | A homolog of C-type lectin |
|  | DP71L | Reducing the activity of p53 |
| Induce apoptosis | E183L (P54) | Interacting with the light chain of dynein (DLC8) |
|  | A238L | IκB-like molecular, interfering with NF-κB activation; Inhibiting calcineurin phosphatase |
| Inhibit IFNs production | MGF-360 (A276R) | Inhibiting the induction of IFN through targeting IRF3, but have no effect on the JAK-STAT signaling pathway |
|  | MGF-505/530 (A528R) | Inhibiting the induction of IFN and the JAK-STAT signaling pathway |
|  | I329L | A homolog of TLR3, inhibiting the activation of IRF3 and NF-κB |
|  | DP96R | Inhibiting type I IFN expression and NF-κB by inhibiting TBK1 and IKK-β |
|  | MGF-360-12L | Inhibiting Type I Interferon Production by Blocking the Interaction of Importin α and NF-κB Signaling Pathway |
|  | pMGF-505-7R | Inhibiting the cGAS-STING signaling pathway by degrading STING |
| Inhibit autophagy | A179L | Interacting with Beclin-1 |
|  | E199L | Promoting cell autophagy through the interaction of PYCR2 |
| Inhibit MHC-I expression | EP153R | Interacting with MHC-I via lectin domain and impairing MHC-I exocytosis |
| Inhibit IL-1β | pL83L | Interacting with IL-1β |

**Responses to Reviewer #3:**

**Summary**

The authors study the role of MGF505-7R in modulating IL-1β and type I IFN response in porcine alveolar macrophages (PAMs) and during infection in swine. Authors showed the pattern of cytokines expression (by ELISA and qRT-PCR) in PAMs infected with ASFV. They then demonstrated the inhibitory effect of ASFV infection in the IL-1β and type I IFN response in PAMs treated with different inducers (as LPS and polydA:dT). Following this, they showed the inhibitory effect of MGF505-7R in IL-1β and type I IFN production in HEK293T cells transfected with 15 different ASFV MGF genes and the corresponding reporter systems. MGF505-7R was then shown to inhibit NF-κB, and IFNα/β activation in a dose fashion in HEK293T cells transfected with the corresponding genes and reporter systems. A virus lacking MGF505-7R gene (ASFV-Δ7R) induced higher levels of IL-1β and IFNβ when infecting PAMs than the wild type (WT) ASFV does. Following this, siRNA specific for TLRs inhibit the increased levels of IL-1β in ASFV-Δ7R infected PAMs. Also, MGF505-7R over expression inhibits NF-κB activation provoked by LPS, IKKα or IKKβ, and IKKα, and MGF505-7R co-precipitate with IKKα when constructs encoding them were co-transfected. Also, PAMs transfected with siRNA specific for NLRP3 gene decreased the IL-1β expression during ASFV-Δ7R infection and co-precipitation of MGF505-7R and NLPR3 were shown to occurs in HEK293T cells transfected with both genes. In addition, over expression of MGF505-7R inhibit IFNβ activation promoted by different inducers (including IRF3), and cells co-transfected with the corresponding genes were used to co-precipitate MGF505-7R and IRF3. In addition, nucleus translocation of IRF3 is halted by expression of MGF505-7R. ASFV-Δ7R showed an attenuated phenotype compared to the WT virus. ASFV-Δ7R infected animals presented a delayed presentation of the disease, at 60% survival, and significantly less virus genome than those infected with the WT virus. In addition, ASFV-Δ7R infected animals presented decreased levels of IL-1β and IFNβ.
The manuscript provides evidence of the function of ASFV gene MGF505-7R indicating its role in IL-1β and IFNβ down expression, describing mechanisms for the modulation of the expression of both genes and showing the involvement of MGF505-71R gene in virulence during pig infection.
The importance of ASFV as an etiological agent of the enormous pandemic currently affecting pig production in Eurasia make the study of ASFV genes important towards the generation of information that may allow for the development of effective virus countermeasures.
ASFV MGF genes have been already described as clearly responsible for down regulating the innate immune response (particularly type-I IFN) during the infection in pigs (reviewed in ref. 4). Several virus genes belonging to the MGFs have been implicated in the down modulation of the host immune response, particularly type I IFN (refs 18, 19, 21, 24, doi.org/10.3390/v13020255).
Very importantly for this manuscript, Li et al. (doi/10.4049/jimmunol.2001110) recently demonstrated the role of the same gene, MGF505-7R, in down modulating host IFNβ response and involved in the process of virus virulence during the infection in pigs. As in the manuscript under review, manuscript Li et al., described a mechanism of how MGF505-7R down regulates IFNβ response in vitro cell cultures and how an ASFV lacking MGF505-7R gene showed an attenuated phenotype in pigs inducing higher host IFNβ response than the WT virus does. Differences reside in that the current report presents the case that MGF505-7R targets specific adaptor proteins (TLRs, NF-kB and NLRP3) of the innate immunity signaling pathway to inhibit the expression of IL-1β and type I IFNα/β while Li et al., indicated that MGF505-7R targets the cGAS-STING cystosolic DNA sensing pathway which function upstream from NF-kB activation. Both reports make the case that MGF505-7R regulates different sensing and adaptor proteins that ultimately lead to the production of IFN. Also, using different methodologies both reports demonstrated that overexpression of MGF505-7R inhibits the translocation of IRF-3 to the nucleus.

**Question 1:** In my opinion, the strong parallelism between results already published by Li et al., and the manuscript under review severely affects the novelty of the results reported in the later.

Authors’ response:

During review of this manuscript, the paper (Li *et al.*, J Immunol, 2021) characterizing the role of ASFV MGF-505-7R in negatively regulating cGAS-STING pathway by promoting ULK1-mediated STING degradation was published. Both researches represent totally independent work. Although Li *et al.* also characterized ASFV MGF-505-7R as a key negative regulator of the host innate immune system and identified the underling mechanism, it does not compromise the novelty of the paper too much as we uncovered totally different mechanism by which ASFV antagonizes IL-1β and type I IFN production. Our data, beyond those previously published, still have obvious novelties, including the following aspects:

1. Inflammatory factors are key components of host innate immunity and can pose a strong anti-viral effect against viruses. IL-1β, a potent pleiotropic proinflammatory cytokine produced predominantly by monocytes, macrophages, and lymphocytes, plays pivotal roles in regulating innate immune responses and instructing adaptive immune responses. In the study, we found that ASFV infection induces low levels of IL-1β production through TLRs/MyD88 pathway and NLRP3 inflammasome in PAMs (Fig. 1, Fig. S4 and Fig. S6). Also, we found that ASFV antagonizes IL-1β production even in the presence of strong inducers such as LPS and Nigericin for IL-1β and type I IFN. To explore the underling mechanism, we screened members of pMGFs for regulation of IL-1β production and found that pMGF505-7R shows the strongest inhibitory effect (Fig 3A). Furthermore, we demonstrated that pMGF505-7R interacts with the IKKα complex to inhibit NF-κB activation, leading to reduced pro-IL-1β transcription and production (Fig1, 4 and 6); meanwhile, pMGF505-7R binds to NLRP3 to inhibit NLRP3 inflammasome assembly, leading to decreased IL-1β production (Fig 7). These results are reported for the first time so far.

2. Type I IFNs are critical host immune mediators for inhibiting viral infections. In this study, we found that ASFV infection induces low levels of type I IFNs in PAMs and pigs (Fig. 1 and 10). Mechanistically different from the one reported by Li et al. in which pMGF505-7R directly targets STING for degradation in the cGAS-STING signaling pathway by recruiting ULK1, in this research we showed that pMGF505-7R inhibits type I IFNs production by directly binding to IRF3 to block its nuclear translocation (Fig. 8). These data also represent novel discovery of the mechanism by which ASFV utilizes to block type I IFN signaling.

3. In animal experiment, Li *et al.* showed that the virulence of ASFV lacking MGF505-7R (ASFV-Δ7R) is dramatically reduced and it is safe to piglets. This conclusion may mislead the researchers who are designing attenuated live vaccines (ALVs) for ASFV as our data showed that ASFV-Δ7R still has moderate virulence. Therefore, it is more urgent to publish our results to provide more information about ASFV studies. In our study, pigs were challenged intramuscularly (i.m.) with ASFV-Δ7R or ASFV-WT at a dosage of 10^3^ or 10^5^ HAD_50_ (Fig 9). The body temperatures of all animals increased gradually, peaked at 41.7°C by day 9 (Fig 9A), and over 60% of pigs challenged with the attenuated ASFV-Δ7R survived (Fig 9B). It is noteworthy that the viral DNA copies in the ASFV-Δ7R-inoculated pigs decreased 2 to 3 orders of magnitude (Fig 9C). We also found that the attenuated ASFV-Δ7R induced higher levels of type I IFN and IL-1β production in pigs (Fig 10), which may help explain the decreased virulence of ASFV-Δ7R. These data provide more detailed information for attenuated ASFV-Δ7R, which may help researchers for designing effective vaccines against ASFV.

**Question 2:** Lines 136-163: these “introductory” results showing that ASFV infection alter the expression of cytokines in PAMs could be deleted since they are not novel nor have incidence in the results presented later.

Authors’ response:

The “introductory” results showing that ASFV infection alters the expression of cytokines in serum of infected-pigs. There is no systematic research data about cytokines in ASFV infected-PAMs, including IFN and inflammatory factors. The study of inflammatory factors induced by ASFV infection in PAMs and the conclusion that ASFV induced low level of inflammatory cytokines compared with other viruses is novel. Based on these data which suggest that ASFV infection actually suppresses inflammatory and interferon responses, we screened the viral proteins for affecting IL-1β and IFN-β and conducted the in-depth research shown in the figures. Therefore, we think that these data are important and necessary.

**Question 3:** Lines 199-208: ASFV-Δ7R is an essential tool in this manuscript therefore the development of recombinant ASFV-Δ7R should be better described. Was the MGF505-7R gene partially/completely deleted? May the deletion compromise adjacent genes? Authors should report the full length sequence of virus genome to ensure the absence of unwanted mutations elsewhere in the genome.

Authors’ response:

Thank you for your suggestion.

(1) We have revised the description of the recombinant ASFV-Δ7R and related methods in Materials and Methods section. To be clear, the complete MGF505-7R gene was deleted and replaced by a cassette containing the fluorescent gene *egfp* under the control of ASFV p72 promoter.

(2) We had sequenced the whole genome of ASFV-Δ7R, and found that none of other genes (including the adjacent genes) were affected except the MGF505-7R deletion and replacement with the cassette. In addition, we analyzed the transcriptome of PAMs infected with ASFV-Δ7R and ASFV-WT and found that the mRNA levels of other MGF genes did not change except MGF505-7R.

**Question 4:** Lines 241-242: authors indicate that MGF505-7R expression inhibits NF-κB activation provoked by IKKα in a dose dependent manner. Figure 6B does not show a dose dependent response. The same is true regarding the inhibitory effect of MGF505-7R expression on the activation of IFNβ promoter by IRF3-5D (lines 290-298 and Figure 8C).
Authors’ response:

According to your suggestion, we repeated the experiment and obtained the same results showing better dose-dependent inhibitory effects of MGF505-7R on NF-κB activation provoked by IKKα and IFNβ activation by IRF3-5D. Please see the Fig. 6B and Fig. 8C in the revised manuscript.

**Question 5:** All immune co-precipitations presented in the manuscript are made in target cells transfected with plasmids encoding the proteins under study. Usually, levels of protein expression achieved by this procedure are not physiological; therefore, it may not represent what actually happens in the infected cells. Authors should mention this issue and, also, try to confirm that the reported interaction between proteins indeed occurs during the ASFV infection.

Authors’ response:

Thank you for your suggestion. I totally agree with you. We had tried to prepare antibody against pMGF505-7R, but the obtained antibody was not good enough for immunoprecipitation assay.

In order to confirm the interaction between ASFV MGF505-7R and endogenous proteins (IRF3, IKKa and NLRP3) during the ASFV infection, we generated a genetically modified ASFV in which an EGFP tag was inserted at the N-terminus of the MGF505-7R gene locus, and named it as ASFV-EGFP-7R (Fig. S5). The expression of EGFP-7R was confirmed by EGFP antibody and the functions of pMGF505-7R seemed to be not affected by the N-terminal fusion of EGFP (Fig. S5F-G). Using the ASFV-EGFP-7R, we confirmed the interactions between ASFV MGF505-7R and endogenous proteins (IRF3, IKKa and NLRP3) in the condition of ASFV-EGFP-7R infection (Fig. 6E, 7C and 8F). Please see the updated results in the revised manuscript.

**Question 6:** Lines 301-306: confocal studies showing the effect of MGF505-7R on IRF3 translocation in PAMs must be confirmed by comparing the differential presence of IRF3 translocation in cells infected with ASFV WT or ASFV-Δ7R.

Authors’ response:

According to your suggestion, PAMs were infected with ASFV-WT or ASFV-Δ7R and the the effect of MGF505-7R on IRF3 translocation was confirmed by comparing the differential presence of IRF3 protein in nuclear and cytoplasm. Please see Fig. 8H the revised manuscript.

**Question 7:** Lines 325-342: determinations described here probably were performed in the same group of animals already described in lines 307-324. If they are the same animals, are panel C in Figure 9 and panel D in Figure 10 showing the same results?

Authors’ response:

The panel C in Figure 9 (Fig. 9C) and panel D in Figure 10 (Fig. 10D) were performed in the same group of animals. The samples in Fig. 9C were different tissue samples collected from all the survived pigs at day 19 when they were euthanized, and the result reflects distribution and levels of viruses in these different tissues of the survived pigs following ASFV-WT or ASFV-Δ7R infection. While the samples shown in Fig. 10D were serum samples collected separately at day 1, day 5 and day 8 post ASFV-challenge from inoculated pigs and the result reflects the dynamics of virus replication and the difference between ASFV-WT and ASFV-Δ7R replication. The two results provide different information concerning the viral load. We provided more information in the related Figure Legends section. Please see the changes (lines 954-955) in the revise manuscript.

**Minor Issues:**

None
